# Applications and Limitations of Inflammatory Biomarkers for Studies on Neurocognitive Impairment in HIV Infection

**DOI:** 10.1007/s11481-013-9512-2

**Published:** 2013-11-21

**Authors:** Edana Cassol, Vikas Misra, Susan Morgello, Dana Gabuzda

**Affiliations:** 1 Dana Farber Cancer Institute, Harvard Medical School, Boston, MA USA; 2 Mount Sinai Medical Center, New York, NY USA; 3 Dana Farber Cancer Institute, 450 Brookline Avenue CLS 1010, Boston, MA 02215 USA

**Keywords:** HIV, HCV, HIV-associated neurocognitive disorders, Innate immune activation, Inflammatory biomarkers, Interferon-α, IL-6

## Abstract

Despite reduced prevalence of severe forms of HIV-associated neurocognitive disorders (HAND) on current antiretroviral therapy (ART) regimens, milder forms of neurocognitive impairment (NCI) remain prevalent in HIV-infected populations. These mild forms of HAND consist of subtypes, probably reflecting distinct, though possibly overlapping, pathophysiological mechanisms. Factors associated with HAND in HIV patients with prolonged viral suppression on ART include older age, low nadir CD4, active HCV co-infection, and cardiovascular risk factors, but underlying mechanisms and their relationship to innate immune activation, chronic inflammation, and other features of systemic disease are poorly understood. In this article, we discuss applications and limitations of plasma inflammatory biomarkers for studies on HAND in HIV patients on ART and describe an analysis pipeline to reduce common sources of noise and increase likelihood of identifying relevant inflammatory biomarkers. Clinical covariates and comorbidities that influence inflammatory biomarkers, such as aging, obesity, metabolic abnormalities, HCV co-infection, and substance abuse, are also reviewed. As an example for using this analytic pipeline, we present an exploratory study of 22 plasma inflammatory biomarkers (IFN-α 2b and -γ, 16 cytokines/chemokines, sIL-2R, sCD14, HA, and YKL-40) in a cohort of HIV-infected individuals with advanced disease, frequent HCV co-infection, and viral suppression on ART. The identification of inflammatory biomarkers associated with HAND in HIV+ patients on ART may be useful to distinguish between HAND subtypes with distinct pathophysiology, and is important for achieving a systems-level understanding of the biology of these disorders, developing effective therapies, and evaluating therapeutic outcomes.

**Electronic supplementary material:**

The online version of this article (doi:10.1007/s11481-013-9512-2) contains supplementary material, which is available to authorized users.

## Introduction

HIV-associated neurocognitive disorders (HAND) range from asymptomatic neurocognitive impairment (ANI) to minor neurocognitive disorder (MND) and HIV-associated dementia (HAD), affecting 20-50 % of HIV-infected individuals despite viral suppression on combination antiretroviral therapy (ART) (McArthur and Brew 2010; Sacktor et al. 2002; Tozzi et al. 2005; Letendre et al. 2010; McArthur et al. 2010; Heaton et al. 2011). HIV infects macrophages/microglia in the central nervous system (CNS), and infection and activation of the monocyte/macrophage system is associated with more severe forms of HAND (i.e., MND and HAD) in untreated patients (Gras and Kaul 2010; Ryan et al. 2001; Gartner and Liu 2002; Gonzalez-Scarano and Martin-Garcia 2005; Kaul et al. 2005; Ancuta et al. 2008). Less is known about mechanism(s) underlying the mild forms of HAND that are now prevalent in patients on ART with viral suppression in plasma and cerebrospinal fluid (CSF). Furthermore, these mild forms of HAND appear to have subtypes, probably reflecting distinct, though possibly overlapping, pathophysiological mechanisms. These mechanisms are not yet defined, but may include dysregulated innate immune responses and chronic inflammation associated with persistently elevated type I and II interferons, ongoing low-level viral replication, vascular injury, metabolic abnormalities, glial cell dysfunction, decreased white matter integrity, and adverse effects of ART drugs (Brew and Letendre 2008; Ances et al. 2009; Everall et al. 2009; Borjabad et al. 2011; Harezlak et al. 2011; Heaton et al. 2011; del Palacio et al. 2012; Gelman et al. 2012a, b; Robertson et al. 2012; Wright et al. 2012).

Biomarkers are objective and measurable features detected in biological fluids (i.e. plasma, urine, CSF) that reflect pathogenic processes, disease progression, and therapeutic responses (Naylor 2003; Mayeux 2004). Commonly used in epidemiological studies to assess risk and in clinical trials as endpoints, biomarkers can also provide mechanistic insights into the biology of disease. Although potential biomarkers have been proposed, there are no specific biomarkers of HAND in plasma or CSF from HIV patients on suppressive ART and the accepted standard for HAND diagnosis is based primarily on standardized screens to assess symptoms and functional impairment, along with neuropsychological testing and excluding other causes of neurocognitive dysfunction (reviewed in (Schouten et al. 2011; The Mind Exchange Working Group 2013)).

Despite the success of ART, chronic inflammation continues to be a hallmark of HIV infection that predicts disease progression and adverse clinical events (Ortiz and Silvestri 2009; d’Ettorre et al. 2011). Although markers of immune activation were associated with more severe forms of HAND in the pre-combination ART era (Brew et al. 1997; Ellis et al. 1997; McArthur et al. 1997; Enting et al. 2000; Ryan et al. 2001; Gartner and Liu 2002; Sacktor et al. 2002), it has been difficult to identify inflammatory biomarkers associated with milder forms of HAND in HIV patients with prolonged viral suppression in the post-combination ART era (The Mind Exchange Working Group 2013). One explanation for this difficulty is the likely contribution of non-inflammatory mechanisms, such as cerebrovascular dysfunction, metabolic alterations, or neurotoxicity of some ART regimens, to some forms of HAND. Other potential explanations are the lower levels of immune activation in HIV patients with prolonged viral suppression on ART and confounding effects of demographic and disease-related variables (e.g. age, race, gender, nadir CD4 counts, ART regimen, HCV co-infection, heavy smoking, alcohol and illicit drug abuse, etc.), which increase noise in the analytic pipeline and make it more challenging to identify relevant biomarkers. In this article, we discuss potential applications and limitations of using inflammatory biomarkers to understand HAND pathophysiology, and describe an analysis pipeline to reduce sources of noise and increase likelihood of identifying inflammatory biomarkers with biological or clinical relevance. As an example of using this analytic pipeline, we present an exploratory study of 22 plasma inflammatory biomarkers in a cohort of HIV-infected individuals with advanced disease and suppressed plasma viral load (VL) on ART.

## Current state of plasma inflammatory biomarkers in HAND

In the pre-combination ART era, disease markers associated with severe forms of HAND included plasma and CSF HIV RNA and several inflammatory markers linked to innate immune activation (e.g., CCL2, IL-6, soluble CD14 [sCD14], neopterin, kynurenine, quinolinic acid) (Brew et al. 1997; Ellis et al. 1997; McArthur et al. 1997; Enting et al. 2000; Ryan et al. 2001; Gartner and Liu 2002; Sacktor et al. 2002). In post-combination ART era cohorts, however, these associations have not been demonstrated in HIV patients with prolonged viral suppression on ART. For example, increased plasma soluble CD14 (sCD14), a monocyte activation marker, was demonstrated HIV+ patients with advanced disease, non-suppressed plasma VL on ART, and neurocognitive impairment (NCI); however, plasma sCD14 has not been associated with NCI in patients with suppressed plasma VL (Ryan et al. 2001; Lyons et al. 2011; Kamat et al. 2012a). Elevated plasma sCD163, a marker of macrophage activation associated with cardiovascular disease, is one of the only inflammatory biomarkers that has been associated with NCI In HIV+ patients with suppressed plasma VL on ART (Burdo et al. 2013). Other factors associated with HAND in HIV patients on suppressive ART are mainly demographic and disease/comorbidity-related factors, including older age (> age 50), low CD4 nadir, active HCV co-infection, cardiovascular risk factors, and some features of metabolic syndrome (e.g. diabetes and increased waist circumference) (Ellis et al. 2011; Heaton et al. 2011; Devlin et al. 2012; McCutchan et al. 2012; Wendelken and Valcour 2012; The Mind Exchange Working Group 2013; Leeansyah et al. 2013; Rempel et al. 2013; Sun et al. 2013). Further studies are needed to identify relevant biomarkers associated with or predictive for development of HAND in HIV patients with prolonged viral suppression on current ART regimens.

## Potential applications of inflammatory biomarkers for studies on NeuroAIDS

In HIV patients on suppressive ART, systemic immune activation is associated with poor clinical outcomes and increased risk for non-AIDS co-morbidities including cardiovascular, liver, kidney, and bone disease (Carr 2003; Brown and Glesby 2012). The causes of persistent immune activation are poorly understood, and may include low levels of ongoing HIV replication, microbial translocation, co-infections with non-HIV pathogens, loss of immunoregulatory cells, persistent elevation of type I and II IFN, and dysregulated cytokine/chemokine production (Appay and Sauce 2008; Boasso and Shearer 2008; d’Ettorre et al. 2011; Sandler and Douek 2012). Many cellular and soluble immune activation markers have been used to study relationships between immune activation and clinical outcomes in patients on ART. T-cell activation is a strong predictor of morbidity and mortality in untreated HIV patients (Appay and Sauce 2008; d’Ettorre et al. 2011), but plasma markers of innate immune activation, including markers of mononuclear phagocyte activation, are better predictors of mortality and adverse clinical outcomes such as cardiovascular disease in HIV patients on ART (Appay and Sauce 2008; Sandler et al. 2011; Burdo et al. 2013). For example, elevated plasma sCD14 independently predicts all-cause mortality in HIV-infected subjects on ART (Sandler et al. 2011) and the macrophage activation marker sCD163 has been shown to be a predictor of non-calcified coronary plaques (Aristoteli et al. 2006). Additionally, elevated plasma IL-6 has been shown to predict age-related comorbidities including cardiovascular disease and all-cause mortality (Kuller et al. 2008; Rodger et al. 2009). Further studies are required to better understand relationships between innate immune activation and these clinical outcomes.

Despite suppressed plasma viral loads in HIV patients on ART, a substantial proportion of patients continue to show evidence of sustained CNS macrophage/microglial activation and low levels of intrathecal inflammation (Gisolf et al. 2000; Eden et al. 2007; Schouten et al. 2011). Consistent with these findings, chronic activation of IFN-α-driven immune responses has been associated with NCI in HIV patients on ART in some cohorts (Rempel et al. 2010; Borjabad et al. 2011; Pulliam et al. 2011; Gelman et al. 2012a, b; Rempel et al. 2013). The identification of a plasma inflammatory biomarker panel that may distinguish between HAND subtypes with different relationships to systemic and/or CNS innate immune activation in terms of their pathophysiology is important for understanding the biology and classification of these disorders (i.e., caused by inflammatory vs. non-inflammatory mechanisms) and evaluating therapeutic outcomes.

## Practical issues and considerations for inflammatory biomarker studies in HIV+ cohorts

### Strategies to reduce noise in the analysis pipeline

**Fig. 1:**
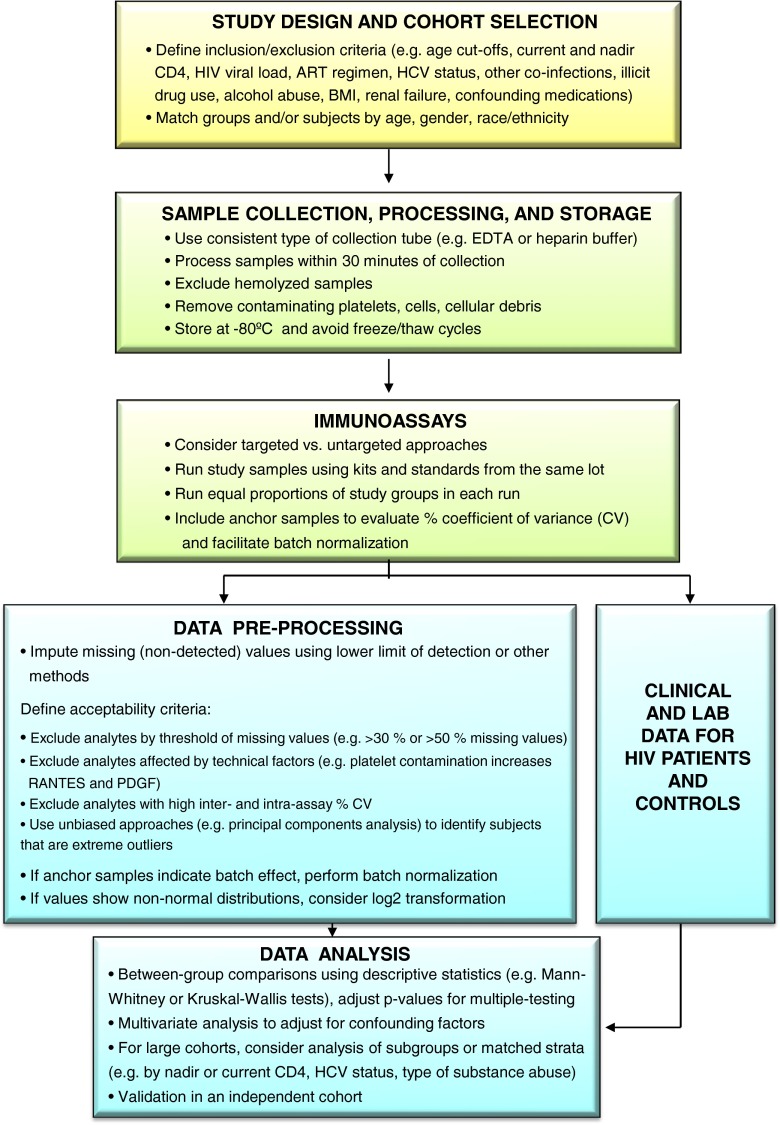
Analysis pipeline to identify plasma inflammatory biomarkers with biological or clinical relevance in HIV patients on ART. Flowchart illustrates workflow to address issues important for inflammatory biomarker study design (*yellow boxes*), sample collection and immunoassays (*green boxes*), and data pre-processing and analysis (*blue boxes*) to reduce sources of noise frequently encountered in inflammatory biomarker studies in HIV-positive cohorts. Addressing these issues in study design, implementation, and data analysis will increase the chance of identifying inflammatory biomarkers with biological or clinical significance. Similar workflows were used in prior studies to identify inflammatory biomarkers associated with neurocognitive impairment and other clinical endpoints in HIV patients on ART (Lyons et al. 2011; Kamat et al. 2012a, b; Cassol et al. 2013)

A major challenge in biomarker studies is limiting potential sources of noise that interfere with the ability to identify valid biomarkers and increase the probability of identifying false positives and false negatives. As such, efforts should be made to reduce sources of noise to optimize study outcomes. A well-designed study cohort and analysis workflow can improve study outcomes by reducing noise in downstream analysis, which increases the probability of identifying relevant inflammatory biomarkers (Fig. 1).

An important first step is selecting well-characterized study populations. In addition to matching groups and/or subjects by age, gender, and race/ethnicity, it is important to consider the following variables during study design: nadir and current CD4 cell counts, plasma HIV VL, type of ART regimen, HCV status (HCV Ab and RNA), alcohol and illicit drug use/dependence (defined by self-report and/or urine toxicology), advanced liver disease, renal failure, and metabolic status (e.g. extreme obesity defined by very high body-mass index [BMI], uncontrolled diabetes defined by high hemoglobin A1c (HbA1c) levels). Several covariates of particular relevance for inflammatory biomarker studies are discussed in greater detail below. For analysis of neurocognitive status, education level is another important variable for matching between groups, or adjustment in multivariate analysis. Given heterogeneity of current HIV cohorts, it is often not possible to match groups or subjects by all potentially confounding factors. Future cohort studies should be designed to collect data for these variables in order to achieve a systems-level understanding of HAND in relation to confounding or contributing factors, including HCV and other co-infections, substance abuse, and functions of other relevant organ systems (e.g., gut, liver, kidney, cardiovascular, and endocrine system). When matching is not possible, clinical data for important covariates should be obtained for HIV patients and controls to characterize the study population. This information can then be integrated into downstream analyses (Fig. 1).

Standardization of sample collection, processing, and storage is also essential to the limit the introduction of potential sources of noise. Blood samples should be collected using consistent methods, processed within 30 min of collection to avoid platelet activation and protein degradation and frozen; freeze/thaw cycles should be avoided. The age and quality of samples should be evaluated prior to running immunoassays, since high variability in storage time or sample quality can increase noise in immunoassay results. Hemolysis, cell debris, and platelet contamination should be avoided because they can interfere with some immunoassays. To minimize batch effects, immunoassays should be run as consistently as possible, using the same lot of kits and standards across the study. For large studies, with samples run on multiple days across multiple plates, anchor samples (e.g., 4–6 identical samples) should be included on every plate to register analytes levels across runs and facilitate batch normalization. Anchor samples should include representative research specimens from each subject group (e.g. healthy controls and HIV-infected subjects) with a range of detectable analyte levels. Batch effects are common, and normalization can help minimize effects of inter-run variability.

Data pre-processing is another important step to reduce noise in data analysis. For analysis of inflammatory biomarkers, we have found that the ability to identify relevant biomarkers is increased by excluding low-quality markers with poor assay performance and a high proportion of missing/non-detected values (e.g., >30 % missing values), analytes known to be affected by confounding technical factors (e.g., platelet contamination increases RANTES and platelet-derived growth factor) and analytes with poor inter- and intra-assay coefficient of variation (e.g., >30 %). However, when the pattern of missing/non-detected values is biologically relevant (for example, when most missing/non-detected values occur in the healthy control group), the data should be evaluated using non-parametric tests and/or by transforming the data into a categorical variable. Subjects identified as extreme outliers (which can reflect technical factors such as poor sample quality or confounding comorbidities such as advanced kidney disease) using unbiased approaches (e.g., data distribution plots, principal components analysis (PCA)) during preliminary analyses to evaluate overall data architecture, are considered as possible candidates for exclusion from downstream analyses. However, exclusion of extreme outliers based on unbiased analysis of the overall dataset should be limited to <5 % of the total study cohort to avoid outcome bias. Data analysis should use appropriate statistical methods and adjust for multiple-testing. Bonferroni adjustments for multiple-testing are appropriate for multiple between-group comparisons to reduce Type 1 errors (false positives), but are too stringent for analysis of large ‘omics datasets such as untargeted multiplex, metabolomics, and proteomics profiling due to the high rates of Type 2 errors (false negatives). Instead, it is common practice to control for multiple-testing in multiplex and ‘omics datasets by controlling the false discovery rate (FDR), with acceptable cut-offs typically below 5 % or 10 %.

Finally, it is important to acknowledge limitations of the study. When certain variables are not matched or not available, their potential confounding effects should be discussed. Ideally, biomarker studies should be performed on large homogeneous cohorts. However, in reality this is not always possible. As such, biomarkers should be validated in larger well-defined independent cohorts. Furthermore, biomarker associations identified from cross-sectional studies should be examined in follow-up studies using longitudinal clinical data to assess predictive value.

### Inclusion/exclusion criteria for study design

Factors associated with increased risk of HAND in HIV patients with viral suppression on ART include older age, low CD4 nadir, active HCV co-infection, and cardiovascular risk factors, but the mechanisms underlying HAND and their relationship to systemic inflammation are poorly understood. To identify inflammatory biomarkers that may provide mechanistic insights into the biology and pathogenesis of HAND, well-designed biomarker studies should exclude or account for non-HIV causes of inflammation as discussed below.

#### Effects of aging on inflammatory biomarkers

Recent studies suggest that HIV may interact with age, particularly in populations over age 50, increasing the prevalence of cognitive impairment in older individuals (Goodkin et al. 2001; Justice 2010). However, the strength of this HIV-age interaction, and age cut-off when it becomes most significant (i.e. over age 50 vs. over age 60), is still debated (Wendelken and Valcour 2012). Moreover, HIV-age interactions may be influenced by a number of clinical covariates, such as duration of HIV infection, prolonged exposure to certain drugs used in ART, type of ART regimen, heavy smoking, patterns of substance abuse (e.g. heavy chronic use of alcohol, cocaine, methamphetamine, or nitrite inhalants), obesity, and HCV co-infection. While mechanisms underlying interactions of HIV infection with aging are poorly understood, they may relate in part to the known effects of aging on chronic inflammation. Chronic low-grade inflammation is a common manifestation of aging, an effect often referred to as “inflammaging”, and increased circulating levels of inflammatory biomarkers such as IL-6, TNF, C-reactive protein (CRP), and serum amyloid A, have been detected in elderly compared to younger subjects, even in the absence of chronic disease (Shaw et al. 2010; Bruunsgaard and Pedersen 2003; Franceschi et al. 2007). Accordingly, when designing biomarker studies, it is important to account for potentially confounding effects of age on inflammatory biomarker profiles and cognitive function. As such, study subjects should be matched for age (within 5 years) and inflammatory biomarkers should be evaluated using approaches that include age as a variable for optimal study design and analytic workflows.

#### BMI, metabolic status, and inflammatory biomarkers

Despite the success of ART in reducing HIV-associated morbidity and mortality, long-term treatment is frequently associated with metabolic abnormalities including dyslipidemia, lipodystrophy, and insulin resistance (Carr 2003; Brown and Glesby 2012). Furthermore, similar to recent trends in the general population, the prevalence of obesity is increasing in HIV patients on ART (Crum-Cianflone et al. 2010). Many of these metabolic abnormalities, which include obesity-induced insulin resistance and type 2 diabetes, are associated with activation of inflammatory signaling pathways, chronic inflammation, and increased risk of inflammatory pathologies (reviewed in (Hotamisligil 2006; Lumeng and Saltiel 2011)). Consistent with these findings, in blinded analyses of inflammatory biomarkers in heterogenous cohorts, we found that very high BMI (>40) and high HbA1C (>6.5) were associated with increased inflammatory biomarker levels in HIV-negative control subjects. Given these considerations, together with known inter-relationships between regulation of metabolism and inflammation, we have used BMI >40 and HbA1c >6.5 as exclusion criteria when selecting subjects for inflammatory biomarker studies in smaller HIV+ cohorts when this data is available. However, given increasing rates of obesity and type 2 diabetes in many control and HIV-infected populations, a limitation of this approach is that these cut-offs limit generalizability of findings. As such, in populations with high rates of obesity and diabetes, inflammatory biomarker studies can consider alternative approaches, such as excluding subjects with BMI or HbA1c levels greater than 3 standard deviations from the mean of the normative population.

#### Importance of HCV and other co-infections as clinical covariates

Due to shared methods of transmission, up to 40 % of HIV-infected individuals are co-infected with HCV (Operskalski and Kovacs 2011; Taylor et al. 2012). While effective ART has significantly improved outcomes in co-infected patients, HCV co-infection can further increase innate immune activation, which in turn is associated with higher risk of neurocognitive impairment (Ryan et al. 2004; Letendre et al. 2005; Morgello 2005; Morgello et al. 2005; Parsons et al. 2006; Brew and Letendre 2008; Hinkin et al. 2008; Martin-Thormeyer and Paul 2009; Cohen et al. 2011; Devlin et al. 2012; The Mind Exchange Working Group 2013; Sun et al. 2013). Furthermore, monocyte activation in HIV/HCV co-infected subjects has been shown to correlate with cognitive impairment even in those with suppressed plasma HIV RNA (Rempel et al. 2010; Sun et al. 2013). Given these findings, it is important to match, stratify, or exclude by HCV status (e.g. positive HCV serology and/or HCV RNA) in studies that seek to identify associations between inflammatory biomarkers and HIV-related NCI. Other chronic co-infections such as Hepatitis B, reactivated Cytomegalovirus, and Tuberculosis, also activate innate immune responses and increase inflammation. As such, occurrence of these co-infections should be matched, accounted for, or excluded, for inflammatory biomarker studies in HIV-infected populations. These approaches will provide a better understanding of similarities and differences in the biology and phenotypes of cognitive impairment associated with HIV mono-infection versus HIV with co-infections, including their relationships to inflammatory biomarkers and innate immune activation.

#### Substance abuse can affect inflammatory biomarkers

Substance abuse continues to be closely associated with the current global HIV epidemic, with an estimated one in three new HIV infections outside sub-Saharan Africa occurring in intravenous drug users (Wolfe et al. 2010). Non-injection recreational drug use is also associated with risk behaviors linked to HIV transmission. Some forms of drug abuse, particularly heavy use of cocaine, methamphetamine, and opioids, have been associated with more rapid disease progression or more severe disease manifestations, including HIV-associated cognitive impairment (Berman et al. 2006; Clark et al. 2013). However, the biological basis for these associations is poorly understood. Substance abuse is associated with decreased ART access and adherence, but stimulants and heroin/morphine also have immunomodulatory effects that can influence inflammation and cognitive function. For example, cocaine abuse may promote inflammation and HIV pathogenesis by causing a Th1-Th2 cytokine imbalance and stimulating IFN-γ responses (Gan et al. 1998; Rios-Olivares et al. 2006; Kamat et al. 2012b). Likewise, methamphetamine has been linked to increased neuroinflammation, which may contribute to its neurotoxic effects (Yamamoto et al. 2010; Clark et al. 2013). In contrast to these pro-inflammatory effects of cocaine and methamphetamine, acute and chronic opioid (i.e., heroin and morphine) administration has immunosuppressive effects, inhibiting humoral and cellular immune responses, natural killer cell activity, cytokine expression, and phagocytic activity (reviewed in (Vallejo et al. 2004)). Opiate effects on neuroinflammation in HIV infection appear to be complex (Byrd et al. 2013). Nonetheless, it is possible to parse deleterious effects of HIV and HIV/HCV co-infection on cognition from substance abuse, given careful attention to clinical/demographic characteristics and methodological rigor (Byrd et al. 2013). Given immunomodulatory effects of illicit drugs, information regarding current and past drug use should be collected when possible, and integrated into the study design and/or data analyses. While data on self-reported drug use is helpful, urine toxicology data is a more reliable measure of recent use and should be used when available to facilitate the evaluation of confounding or amplifying effects of illicit drug use on cognitive impairment.

## Evaluating inflammatory biomarkers associated with HAND: an exploratory analysis in a representative cohort

To date, there has been limited success in using soluble markers of immune activation as inflammatory biomarkers of HAND in HIV patients on suppressive ART, particularly in patients with mild forms of NCI. This may be due in part to confounding effects of demographic and disease-related factors described above, as well as non-inflammatory mechanisms that are likely to underlie some forms of HAND (e.g., cerebrovascular, metabolic alterations, neurotoxicity of some ART drugs, etc.). Previously, we evaluated 17 plasma biomarkers using the Bio-source 25-plex Human Cytokine Assay (Invitrogen) in a cohort of HIV patients with advanced disease and suppressed plasma VL on ART (*n* = 14). We found increased inflammatory biomarkers in plasma and CSF, but none were associated with NCI (Kamat et al. 2012a). However, the groups classified by neurocognitive status (impaired vs. unimpaired) were not well-matched for several important confounding variables.

To identify inflammatory biomarkers associated with HAND in a cohort in which groups classified by neurocognitive status were better matched for confounding variables, we evaluated 22 plasma inflammatory biomarkers (IFN-α 2b and -γ, 16 cytokines/chemokines, sIL-2R, sCD14, HA, and YKL-40) in 30 HIV+ subjects with CD4 nadir <300, high frequency of HCV co-infection, and suppressed VL on ART (<400 plasma HIV RNA copies/ml) and 20 HIV-negative healthy control subjects. We then examined relationships between plasma inflammatory biomarkers and neurocognitive function (assessed by global T scores derived from comprehensive neurocognitive testing at the baseline study visit and after 1 year of follow-up) (Supplemental Methods). The HIV+ subjects were selected to create dichotomous groups (*n* = 15 subjects per group) defined by global T score <40 or ≥40 (corresponding to scores in the impaired or unimpaired range, respectively) matched for age, gender, race/ethnicity, HCV-serostatus, and class of ART regimen (Supplemental Table 1). HIV+ subjects were predominantly male (70 %) and African American (56 %), and at increased risk for HAND due to advanced disease (median current and nadir CD4 counts of 280 and 60 cells/mL, respectively) and high frequency of HCV co-infection (70 %). The group with global T scores <40 was slightly younger (median age 44 vs. 49 years; *p* = 0.06), and had slightly higher current and nadir CD4 counts, but these differences were not significant (*p* = 0.25 and 0.41, respectively).

Although 21 of 22 plasma inflammatory biomarkers were higher in the total cohort of HIV+ compared to healthy control subjects (Supplemental Table 2), when HIV+ subjects were stratified by global T scores at the baseline visit (T score <40 vs. ≥40), no biomarkers distinguished between these groups classified by current neurocognitive status. IL-6 was slightly higher in plasma from subjects with NCI (*p* = 0.089), but this difference was not significant after adjustment for multiple-testing (Supplemental Table 2).

Examining inter-relationships between these 22 biomarkers by Pearson correlation analysis, correlation matrix visualization (Supplemental Fig. 1), and unsupervised clustering on heatmaps (data not shown), identified a cluster of 7 biomarkers (IFN-α 2b, IFN-γ, sIL-2R, IL-1RA, IL-1b, IL-6, and IL-12) that correlated positively in pair-wise comparisons. Previously, we found that continuous descriptors rather than categorical diagnoses are better at identifying associations between biomarker levels and impaired neurocognitive function (Lyons et al. 2011; Kamat et al. 2012a). We therefore performed Pearson correlation analysis to examine relationships between global T scores and inflammatory biomarkers within this correlated cluster. This approach revealed weak inverse correlations between plasma IL-6, IFN-α 2b, and sIL-2R and global T scores in unadjusted univariate models (sIL-2R at *p* < 0.05 and IFN-α 2b and IL-6 *p*-values 0.05 < *p* < 0.1) (Supplemental Fig. 1).

**Fig. 2:**
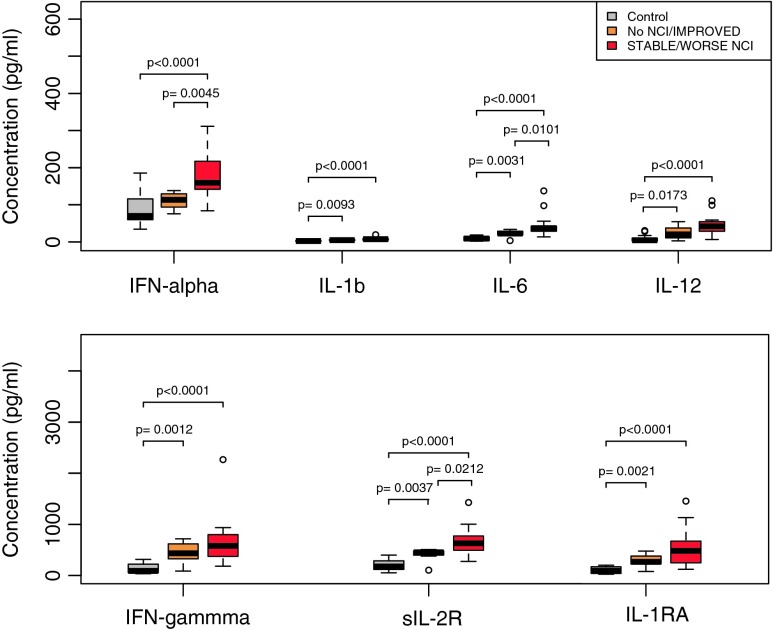
Increased IFN-α 2b, IL-6, and sIL-2R in plasma from HIV+ subjects on ART with stable or progressive neurocognitive impairment (NCI) versus unimpaired neurocognitive status after 1 year follow-up. Shown are boxplots for 7 biomarkers identified as highly correlated features by Pearson correlation analysis (Supplemental Fig. 1). These 7 biomarkers also clustered in heatmaps generated by unsupervised methods. Box plots show inflammatory biomarker levels in healthy controls (*grey*), and HIV subjects with no/improved NCI (*orange*) or stable/worse NCI (*red*) after 1 year follow-up. Medians are represented by horizontal bars, boxes span the interquartile range (IQR), and whiskers extend to extreme data points within 1.5 times the IQR. Outliers are plotted as open circles and lie outside 1.5 times IQR. P-values were calculated using the Mann–Whitney *U* test and adjusting for multiple comparisons by controlling the false discovery rate (FDR) using p.adjust in R. (*P* < 0.05 and FDR < 5 %) (Supplemental Table 3)

Based on follow-up neurocognitive data (HAND clinical diagnoses and neurocognitive test scores) available for 23 HIV+ subjects at 6 and 12 months after the baseline study visit, we then re-classified HIV+ subjects into groups defined by neurocognitive status 1 year later (see Supplemental Methods): no NCI/Improved when HAND clinical diagnoses and neurocognitive test scores indicated unimpaired or improved neurocognitive function (*n* = 7) and stable/worse NCI when subjects remained stably impaired, were unimpaired at baseline and became impaired, or were impaired already and had further decline (*n* = 16; 9 stably impaired and 7 with decline). The stable/worse NCI group had similar age and current or nadir CD4 counts (*p* = .13, .45, and .38, respectively), but higher frequency of HCV co-infection (80 % vs. 50 %), compared to the no NCI/improved group. Comparing biomarker levels between these groups and healthy controls showed that IFN-α 2b was the top-ranked biomarker that distinguished between groups with no or improved NCI versus stable or worse NCI in univariate models adjusted for multiple comparisons by controlling the FDR (Fig. 2 and Supplemental Table 3; *p* < 0.05 and FDR < 5 %). The other biomarkers that distinguished these groups were IL-6 and sIL-2R (*p* < 0.05 and FDR < 5 %). The association between these 3 inflammatory biomarkers and impaired neurocognitive function was detectable only when neurocognitive status was characterized using longitudinal data, probably reflecting more accurate classification of neurocognitive status based on longitudinal data and within-subject trajectories.

Persistent elevation of IFN-α is a major driver of chronic innate immune activation in HIV infection, and IFN-α subtype 2b is the predominant form increased in HIV patient plasma (Lehmann et al. 2009; Hardy et al. 2013). Prior studies identified links between chronic type I interferon responses and HAND in HIV patients on suppressive ART based on monocyte or brain tissue transcriptome analyses (Rempel et al. 2010; Borjabad et al. 2011; Pulliam et al. 2011; Gelman et al. 2012a, b; Rempel et al. 2013). Furthermore, recombinant IFN-α administration in mouse models is associated with neurotoxicity (Sas et al. 2009; Fritz-French and Tyor 2012). Cohen et al. (Cohen et al. 2011) and Pederson et al. (Pedersen et al. 2013) previously demonstrated weak associations between increased plasma IL-6 and impaired neurocognitive function in HIV patients on ART; one of these cohorts (Cohen et al. 2011) had HIV/HCV co-infection. To our knowledge, plasma soluble IL-2 receptor, a marker of T cell activation, has not been previously linked to HAND. Recombinant IFN-α treatment increases plasma IL-6, along with IL-1RA, levels in humans (Tilg et al. 1993; Cotler et al. 2002). Elevated IFN-α 2b, IL-6, and sIL-2R may be indicative of increased innate immune activation along with its stimulatory effects on T-cell activation. Accordingly, our exploratory study identified potential links between markers of innate immune activation and NCI in HIV-infected and HIV/HCV-coinfected patients on suppressive ART.

By unsupervised hierarchical clustering, we found that HIV+ subjects clustered according to levels of plasma IFN-α 2b and IL-6; one cluster consisted of 11/16 HIV+ subjects with stable impairment or worse NCI 1 year later with high levels of these biomarkers at the baseline visit, while the 5 remaining subjects had low levels only slightly above those of uninfected controls (data not shown). Although the small sample size limits conclusions that can be drawn, this preliminary finding, together with previous studies suggesting increased IFN-α-driven innate immune responses in HAND pathogenesis and neurotoxicity induced by recombinant IFN-α administration in mouse models (Sas et al. 2009; Rempel et al. 2010; Borjabad et al. 2011; Pulliam et al. 2011; Fritz-French and Tyor 2012; Gelman et al. 2012a, b), suggests these biomarkers may be useful to distinguish between HAND subtypes that may have different relationships to systemic inflammation. Further longitudinal studies of larger cohorts of subjects on suppressive ART are needed to determine relationships of these biomarkers to future risk of HAND, their utility as biomarkers to distinguish between HAND subtypes with different underlying mechanisms, and their relationship to HIV mono-infection versus HIV/HCV co-infection.

Limitations of this small exploratory study include the small sample size, which limits the power to detect some significant associations and precludes multivariate analysis to evaluate potential confounding effects of low CD4 nadir, HCV co-infection, or differences in ART regimens. The narrow selection criteria used to define the study cohort (CD4 nadir <300) also limits our findings to HIV- or HIV/HCV-infected individuals with advanced HIV disease. Our cohort had a high frequency of HCV co-infection (70 %), which may influence associations between inflammatory biomarkers and NCI (Cohen et al. 2011; Rempel et al. 2013; Sun et al. 2013). As such, we cannot exclude the possibility that HCV co-infection was an important factor driving innate immune activation and/or NCI. Furthermore, we were unable to classify our subjects as “active” or “inactive/past” HCV co-infection due to a lack of available HCV viral load data. A subset of inflammatory biomarkers in the 27-plex array was excluded from our analysis because they did not meet acceptability criteria (e.g., large numbers of missing values, large coefficients of variation, etc.). In the current exploratory study, we used plasma samples to identify inflammatory biomarkers associated with HAND. Plasma biomarkers mainly reflect levels of systemic inflammation. Future studies should examine CSF samples to identify biomarkers of intrathecal inflammation and their relationship to HAND. We included NPI-O subjects, as many likely exhibit neurocognitive deficits attributable to HIV, and there is site-to-site variation in assigning this diagnosis. In past studies, we found that continuous descriptors of neurocognitive status (global T scores) are more sensitive indicators of neurocognitive impairment than categorical diagnoses. However, classification of NCI based on global T score does not always match HAND clinical diagnoses. The present study cohort was from NNTC, which specifically recruits individuals with advanced disease, and CHARTER, which includes a large population of well-controlled HIV+ subjects, to represent a diverse popuation of HIV-infected individuals. As such, the study cohort reflects a bias of urban cohorts with large populations of advanced disease or HCV co-infected patients and results cannot be generalized to all populations. Most of the samples from the NNTC and CHARTER studies were collected in the mid- to late-2000s and may not reflect HIV subjects commonly seen in the clinic today. Further studies are needed to investigate larger, more recent cohorts with less advanced disease, with active vs. inactive vs. without HCV co-infection, treated with specific ART regimens, and stratified by age (i.e. < age 50 vs. > = age 50).

## Conclusions and future perspectives

In studies to date, no single inflammatory biomarker has proven valid among independent cohorts for reflecting the pathobiology of HAND, correlating with its neurocognitive manifestations, or predicting its future development in HIV patients with prolonged viral suppression on ART. Based on the complexity of HAND pathobiology, multifactorial nature of factors contributing to development of NCI in HIV patients on suppressive ART (e.g. aging, metabolic status, co-infections, illicit drug use, etc.), and heterogeneity of current HIV cohorts (e.g. current and nadir CD4 count, initiation of ART, and past and current ART regimens), it may not be possible to identify a single inflammatory biomarker for these applications. Instead a panel of biomarkers is more likely to address these important goals in future studies on HAND. Recently, we identified a plasma inflammatory biomarker signature consisting of CXCL9, CXCL10, sIL-2R, and sCD14 that may be useful as a surrogate marker to monitor immune activation in both viremic and aviremic HIV patients on ART during disease progression and therapeutic responses (Kamat et al. 2012b). Likewise, the exploratory analysis in the present study identified plasma IFN-α 2b, IL-6, and soluble IL-2 receptor as inflammatory biomarkers that merit further study to understand their relationship to neurocognitive impairment, innate immune activation, and HCV co-infection in HIV-infected individuals. Untargeted approaches, such as multiplex assays, are more likely to identify relevant inflammatory biomarkers by screening more candidates, and thereby increasing the likelihood of identifying unexpected associations. Moving forward, the integration of inflammatory biomarkers with data from other high-throughput technologies, such as proteomics and metabolomics, will be important next steps toward the goal of identifying biomarker profiles with biological, clinical, and prognostic relevance. Using a similar approach, in a recent study we integrated inflammatory biomarker and metabolite profiles with clinical variables and obtained novel insights into complex relationships between altered lipid metabolism, hepatic dysfunction, inflammation, and microbial translocation in HIV patients on PI-based ART (Cassol et al. 2013). Thus, when integrated with other ‘omics and clinical data, inflammatory biomarkers are likely to be important for obtaining a systems-level understanding of HAND pathogenesis in relation to systemic inflammation and functional decline of other organ systems in HIV-infected individuals, and may also be useful for classification of HAND subtypes and evaluation of new therapies.

## Electronic supplementary material

Below is the link to the electronic supplementary material.

Supplemental Methods (DOC 53 kb)

Supplemental Table 1 Demographic and clinical characteristics of HIV+ subjects stratified by global T scores and HIV-/HCV- healthy controls in the study cohort. (DOC 45 kb)

Supplemental Table 2 Exploratory analysis of plasma inflammatory biomarker levels in HIV+ subjects classified by neurocognitive status at the baseline visit. Plasma biomarker levels were measured in 50 subjects using multiplex assays or ELISA and compared between HIV+ subjects classified by HIV status and global T scores (< 40 vs. ≥ 40) at the baseline visit and uninfected healthy controls. For the majority of biomarkers tested, levels were higher in the total cohort of HIV+ subjects (n=30) compared to healthy controls (n=20) (Mann–Whitney test, with Bonferroni adjusted threshold p<0.017). P-values for groupwise comparisons in groups defined by neurocognitive status at the baseline visit are highlighted with color codes shown in the legend to indicate p-values corresponding to different thresholds. (PDF 90 kb)

Supplemental Table 3 P-values and false discovery rates (FDR) for between-group comparisons of plasma inflammatory biomarker levels in HIV+ subjects classified by neurocognitive status one year after the baseline visit. Based on HAND clinical diagnoses and global T scores after one year follow-up compared to the baseline visit, HIV+ subjects were classified into dichotomous groups with no/improved neurocognitive impairment (NCI) (n=7) or stable/worse NCI (n=16) at one year follow-up as described in the Supplemental Methods. A third group consisted of HIV-/HCV- healthy controls (n=20). These 7 inflammatory biomarkers represent a cluster of biomarkers that correlated positively in pair-wise Pearson correlations in exploratory analyses of 22 biomarkers (see Supplemental Figure 1). Significant differences were defined by a p-value <0.05 and controlling the FDR at < 5%. FDR was calculated in R using p.adjust. (PDF 10 kb)

Supplemental Figure 1 Pearson correlation matrix visualization reveals inter-relationships between inflammatory biomarkers and global T-scores in HIV+ subjects with advanced disease on suppressive ART. Inflammatory biomarkers were compared in a pair-wise manner and the correlation matrix was constructed in R. Diagonals display the histogram, kernel density curve (purple), and rug for each individual biomarker. Panels to the right of the histogram show Pearson correlation coefficients. The text is scaled relative to the absolute magnitude of the correlation coefficient. P values are represented by purple symbols (*** p<0.001; ** p<0.01; * p<0.05; • p= 0.05-0.1). Panels left of the diagonal show scatterplots. The linear regression line (purple) fits expression values from all 30 HIV subjects. Red and blue circles represent HIV subjects with T scores <40 or ≥ 40, respectively. (PDF 565 kb)
