# Supplementary material for: Applications and Limitations of Inflammatory Biomarkers for Studies on Neurocognitive Impairment in HIV Infection
Source: J Neuroimmune Pharmacol. 2013 Nov 21;8(5):1087–97. doi: 10.1007/s11481-013-9512-2 (PMC3889222; doi:10.1007/s11481-013-9512-2)
Supplement: Supplementary file 1 — (DOC 53 kb) [file 11481_2013_9512_MOESM1_ESM.doc]

**Supplemental Methods**

**Subjects.** Thirty HIV+ subjects (97% with nadir CD4 counts < 300) with samples and data collected between 1999 and 2008 were from 4 sites (Manhattan HIV Brain Bank, National Neurological AIDS Bank, California NeuroAIDS Tissue Network, Texas NeuroAIDS Research Center) within the National NeuroAIDS Tissue Consortium (NNTC) (n= 23) and from CNS Highly Activate Retroviral Therapy Effects Research (CHARTER) (n=7), a six-center observational cohort study. All HIV+ subjects were on suppressive ART (80% on protease inhibitor-based regimens), with plasma HIV RNA < 400 copies/ml. The majority were selected from subjects in the cohort examined in our previous study . To evaluate relationships of plasma biomarkers to neurocognitive test scores, subjects were selected to populate two groups of equal size (n=15 subjects per group) dichotomized by global T-scores <40 versus >40, but matched for similar age, race, gender, % on PI-based ART, and % HCV-seropositive (Supplemental Table 1). Fifteen (50%) had available CSF samples from the same time as plasma samples and neurocognitive testing, all with CSF viral loads < 50 HIV RNA copies/ml. All subjects provided written informed consent under local institutional IRB approval. HAND clinical diagnoses were determined using established criteria based on formal neurocognitive testing and neurological evaluation. Neuropsychological impairment due to other causes (NPI-O) was diagnosed when factors in addition to HIV could contribute to neurocognitive impairment. Subjects with severe psychiatric disorders, a confounding neurological disorder, or active systemic infection were excluded. Twenty plasma samples from healthy donors testing HIV/HCV seronegative were from Bioreclamation LLC (NY) or lab volunteers at Dana-Farber Cancer Institute, obtained with written informed consent and IRB approval.

**Neurocognitive testing.** All HIV+ subjects were administered an identical comprehensive test battery designed to assess seven categories of neurocognitive function (attention, learning, memory, executive function, speed of information processing, motor function, and verbal fluency) . Demographically corrected global T scores (a normalized measure of overall cognitive function derived from formal neurocognitive testing) were generated from the seven individual T scores as described . Global T scores correlate negatively with severity of neurocognitive impairment, with values below 40 signifying impairment (40 corresponds to one standard deviation of 10 from a normalized mean of 50) . In addition to classifying subjects into dichotomous groups based on global T scores >=40 versus < 40 at the baseline visit, subjects were re-classified into the following groups based on HAND clinical diagnoses together with global T scores after one-year follow-up: 1) no NCI/Improved when HAND clinical diagnoses together with neurocognitive test scores indicated unimpaired or improved neurocognitive function (e.g. No NCI remaining No NCI, ANI or MND improving to No NCI); and 2) stable/worse NCI when subjects remained stably impaired, were unimpaired at baseline and then became impaired, or were impaired already and had further decline indicated by worsening HAND diagnosis (e.g. No NCI or ANI progressing to MND, ANI or MND progressing to HAD, etc).

**Biomarker quantification.** sCD14, CCL2, hyaluronic Acid (HA) (R&D Systems), and YKL-40 (Quidel Corporation) were quantified by ELISA. Plasma levels of interleukins (IL-1β, IL-1RA, IL-2RA, IL-4, IL-5, IL-6, IL-7, IL-8, IL-10, IL-12, IL-13), tumor necrosis factor (TNF), interferon (IFN)-γ, IFN-α subtype 2b, CXCL9, CXCL10, CCL3, and CCL4 were measured using a magnetic bead-based multiplex array (Bio-Plex Pro Human Cytokine 27-plex Assay plus sIL-2RA, IFN-α subtype 2b, and CXCL9 single-plex assays).

**Data analysis.** For biomarkers measured by multiplex profiling, missing (non-detected) values were imputed with the minimum value detected for each analyte. When the minimum was extrapolated from the standard curve, the lower limit of detection (LOD) specified by the manufacturer was used. Pre-processing was performed to exclude biomarkers with >30% missing data, >2-fold difference in duplicate samples run across independent experiments, and analytes known to be affected by platelet contamination (i.e., RANTES and platelet-derived growth factor (PDGF)). By this approach, 18 biomarkers in the Bio-plex Assay met acceptability criteria. For an exploratory analysis of these biomarkers, significant differences between groups were evaluated by Kruskal-Wallis (p<0.05) and Mann-Whitney U Test with Bonferroni adjustment for multiple-testing (p<0.017). Pearson correlation coefficients and p-values were calculated using cor.test function in R. False discovery rates (FDR) were calculated in R using p.adjust and controlled at < 5%. Biomarker levels were plotted as a scatter plot matrix using custom scripts based on the PerformanceAnalytics package, and as boxplots using the boxplot function, in R. Hierarchical clustering was performed with dChip software using Euclidean distance and average linkage.

**References**

Antinori A et al. (2007) Updated research nosology for HIV-associated neurocognitive disorders. Neurology 69:1789-1799.

Li C, Hung Wong W (2001) Model-based analysis of oligonucleotide arrays: model validation, design issues and standard error application. Genome Biol 2:RESEARCH0032.

Li C, Wong WH (2001) Model-based analysis of oligonucleotide arrays: expression index computation and outlier detection. Proc Natl Acad Sci U S A 98:31-36.

Lyons JL, Uno H, Ancuta P, Kamat A, Moore DJ, Singer EJ, Morgello S, Gabuzda D (2011) Plasma sCD14 Is a Biomarker Associated With Impaired Neurocognitive Test Performance in Attention and Learning Domains in HIV Infection. J Acquir Immune Defic Syndr 57:371-379.

Woods SP, Rippeth JD, Frol AB, Levy JK, Ryan E, Soukup VM, Hinkin CH, Lazzaretto D, Cherner M, Marcotte TD, Gelman BB, Morgello S, Singer EJ, Grant I, Heaton RK (2004) Interrater reliability of clinical ratings and neurocognitive diagnoses in HIV. J Clin Exp Neuropsychol 26:759-778.
