# Supplementary material for: Applications and Limitations of Inflammatory Biomarkers for Studies on Neurocognitive Impairment in HIV Infection
Source: J Neuroimmune Pharmacol. 2013 Nov 21;8(5):1087–97. doi: 10.1007/s11481-013-9512-2 (PMC3889222; doi:10.1007/s11481-013-9512-2)
Supplement: Supplementary file 2 — Demographic and clinical characteristics of HIV+ subjects stratified by global T scores and HIV-/HCV- healthy controls in the study cohort. (DOC 45 kb) [file 11481_2013_9512_MOESM2_ESM.doc]

|  | HIV-HCV- control subjects (n=20) | HIV+ subjects with Global T score ≥ 40 (n=15) | HIV+ subjects with Global T score < 40 (n=15) | P-value  (HIV+ with Global T score ≥40 vs. <40) |
| --- | --- | --- | --- | --- |
| **Age (years)** |  |  |  |  |
| Mean ± SD | 33 ± 8 | 48 ± 7 | 44 ± 5 | 0.06 |
| Median (range) | 31 (19-47) | 49 (38 - 59) | 44 (36 - 53) |  |
| **Gender** |  |  |  |  |
| Male | 15 (75%) | 12 (80%) | 9 (60%) | 0.43 |
| Female | 5 (25%) | 3 (20%) | 6 (40%) |  |
| **Race** |  |  |  |  |
| African American | 9 (45%) | 9 (60%) | 8 (53%) | 1.00 |
| Caucasian | 8 (40%) | 4 (27%) | 1 (7%) | 0.33 |
| Hispanic | 1 (5%) | 2 (13%) | 6 (40%) | 0.23 |
| **CD4 T cell count (cells/µl)** |  |  |  |  |
| Mean ± SD | NA | 78 ± 77 | 116 ± 147 | 0.41 |
| Median (range) | NA | 59 (2 - 267) | 71 (17 - 536) |  |
| **PI^a^-based ART** | NA | 12 (80%) | 12 (80%) | 1.00 |
| **HCV seropositive** | NA | 10 (67%) | 11 (73%) | 1.00 |
| **HAND^b^ diagnosis** |  |  |  |  |
| No Impairment | NA | 6 (40%) | 0 (0%) | 0.02 |
| ANI | NA | 4 (27%) | 1 (7%) | 0.33 |
| MND | NA | 2 (13%) | 4 (27%) | 0.65 |
| HAD | NA | 2 (13%) | 4 (27%) | 0.65 |
| NPI-O | NA | 1 (7%) | 6 (40%) | 0.08 |

**Supplemental Table 1.** Demographic and clinical characteristics of HIV+ subjects stratified by global T scores and HIV-/HCV- healthy controls in the study cohort.

**^a^**PI, Protease inhibitor; ^b^HAND, HIV-associated neurocognitive disorders; No Impairment, no neurocognitive impairment; ANI, asymptomatic neurocognitive impairment; MND, Mild Neurocognitive Disorder; HAD, HIV-associated dementia; NPI-O, Neuropsychological impairment due to other causes. NA, not available.
