# Supplementary material for: Applications and Limitations of Inflammatory Biomarkers for Studies on Neurocognitive Impairment in HIV Infection
Source: J Neuroimmune Pharmacol. 2013 Nov 21;8(5):1087–97. doi: 10.1007/s11481-013-9512-2 (PMC3889222; doi:10.1007/s11481-013-9512-2)
Supplement: Supplementary file 3 — Exploratory analysis of plasma inflammatory biomarker levels in HIV+ subjects classified by neurocognitive status at the baseline visit. Plasma biomarker levels were measured in 50 subjects using multiplex assays or ELISA and compared between HIV+ subjects classified by HIV status and global T scores (< 40 vs. ≥ 40) at the baseline visit and uninfected healthy controls. For the majority of biomarkers tested, levels were higher in the total cohort of HIV+ subjects (n=30) compared to healthy controls (n=20) (Mann–Whitney test, with Bonferroni adjusted threshold p<0.017). P-values for groupwise comparisons in groups defined by neurocognitive status at the baseline visit are highlighted with color codes shown in the legend to indicate p-values corresponding to different thresholds. (PDF 90 kb) [file 11481_2013_9512_MOESM3_ESM.pdf]

Supplemental Table 2. Exploratory analysis of plasma inflammatory biomarker levels in HIV+ subjects classified by neurocognitive status at the baseline visit. Shown are plasma biomarker levels for all HIV subjects (n=30) and healthy controls (n=20) and between-group comparisons.

Subject ID	Diagnosis	Global T Score (GTS) based Neurocognitive Status	sCD14 (ug/ml)	HA (ng/ml)	YKL-40 (ng/ml)	IL-1b (pg/ml)	IL-1RA (pg/ml)	IL-4 (pg/ml)	IL-5 (pg/ml)	IL-6 (pg/ml)	IL-7 (pg/ml)	IL-8 (pg/ml)	IL-10 (pg/ml)	IL-12 (pg/ml)	IL-13 (pg/ml)	TNF-alpha (pg/ml)	sIL-2R (pg/ml)	CCL2 (pg/ml)	CCL3 (pg/ml)	CCL4 (pg/ml)	IFN-alpha (pg/ml)	IFN-gamma (pg/ml)	CXCL9 (pg/ml)	CXCL10 (pg/ml)
HIV1	NPI-O	GTS ≥40	3.9	217.5	307.7	8.4	455.5	16.6	11.1	25.7	34.9	46.4	17.9	37.8	12.1	136.9	634.7	276.3	7.7	37.9	167.7	612.4	5445.5	9495.1
HIV2	ANI	GTS ≥40	2.8	91.9	115.5	4.8	267.8	7.9	6.7	17.3	20.4	25.4	8.3	20.1	9.6	93.0	416.2	55.4	4.6	31.0	98.1	433.9	1541.8	3941.8
HIV3	ANI	GTS ≥40	2.8	85.9	170.0	7.3	329.5	12.6	9.3	37.1	29.5	49.9	15.6	34.7	21.8	97.3	1058.9	183.7	7.6	143.1	183.4	515.1	5208.5	8973.7
HIV4	MND	GTS ≥40	2.2	117.4	245.3	7.5	512.8	15.6	10.5	36.3	37.0	48.4	36.4	55.7	13.2	150.2	490.8	223.5	6.9	62.8	151.0	758.9	1629.3	1235.8
HIV5	HAD	GTS ≥40	1.9	100.8	420.5	4.2	187.0	7.6	3.7	29.1	17.7	26.3	12.2	20.8	8.9	46.6	361.8	85.8	4.4	72.4	106.7	257.4	2078.8	6049.0
HIV6	ANI	GTS ≥40	1.1	148.3	255.1	11.6	688.7	20.5	17.8	37.0	50.5	58.4	27.5	63.3	17.2	202.1	710.8	176.9	10.4	39.9	211.7	887.2	715.3	1043.5
HIV7	ANI	GTS ≥40	2.1	164.2	181.5	2.9	97.5	4.9	1.8	4.1	9.7	15.6	7.4	36.7	7.4	29.7	206.3	102.4	4.3	24.4	75.9	157.6	651.7	1861.1
HIV8	MND	GTS ≥40	1.4	222.9	127.2	7.1	419.7	12.5	12.3	32.2	32.8	41.5	15.5	33.1	10.4	154.1	498.5	124.5	7.4	34.8	134.7	716.5	710.0	1610.6
HIV9	HAD	GTS ≥40	2.5	140.5	173.2	8.2	703.0	17.6	8.5	38.8	26.6	33.5	59.5	98.7	13.2	158.7	756.4	132.7	5.6	48.1	238.0	689.5	3102.8	4409.0
HIV10	Normal	GTS ≥40	2.3	166.8	88.3	6.3	302.4	12.0	8.4	19.2	26.9	41.8	14.7	50.0	13.0	73.2	487.6	283.9	10.6	86.1	147.8	457.0	1850.1	4114.4
HIV11	Normal	GTS ≥40	2.8	206.4	231.7	3.9	260.9	8.8	2.8	20.5	14.7	31.8	4.7	8.4	15.3	61.7	453.4	149.4	5.2	89.9	113.2	385.6	9196.0	14318.6
HIV12	Normal	GTS ≥40	2.5	109.4	20.0	5.2	222.0	8.9	5.7	16.2	19.8	28.8	9.4	15.6	11.7	57.0	257.2	36.1	4.4	51.9	132.0	358.4	746.9	1593.2
HIV13	Normal	GTS ≥40	2.6	97.4	351.0	5.7	339.8	11.0	5.8	24.5	26.6	39.9	17.0	41.6	11.6	97.0	464.4	238.7	5.3	61.5	124.3	518.2	1885.7	2366.2
HIV14	Normal	GTS ≥40	2.4	97.8	236.5	3.8	198.4	6.2	5.4	13.0	10.9	21.2	15.9	119.5	12.6	62.5	259.4	246.0	4.3	57.9	66.2	314.6	1004.5	2979.0
HIV15	Normal	GTS ≥40	2.0	102.2	181.2	7.3	476.9	14.2	8.7	33.6	36.5	49.1	24.2	54.4	11.0	130.2	506.2		6.8	79.4	138.2	716.8	1560.8	1382.6
HIV16	NPI-O	GTS <40	2.3	283.8	414.9	6.1	401.4	11.6	9.0	25.5	24.7	40.0	14.1	29.2	23.0	90.2	855.8	367.6	6.2	56.1	176.6	482.5	4938.5	9047.2
HIV17	MND	GTS <40	3.1	90.5	195.4	11.1	671.9	20.2	16.2	35.5	49.3	55.1	25.0	47.5	22.0	189.9	663.1	301.2	10.3	59.4	215.8	936.7	661.0	1768.9
HIV18	NPI-O	GTS <40	2.2	243.9	366.5	3.3	123.8	5.6	7.5	55.8	13.3	25.2	8.3	6.5	4.7	39.6	787.2	208.3	1.4	50.0	84.0	190.1	36530.2	5448.3
HIV19	HAD	GTS <40	3.1	153.6	271.3	11.8	675.2	22.5	17.1	37.7	51.5	60.1	27.7	53.5	14.3	194.0	877.5	16.8	10.0	54.6	218.5	923.1	4699.9	8779.7
HIV20	NPI-O	GTS <40	2.0	169.3	150.7	7.7	391.5	13.7	9.7	41.9	31.8	44.6	17.8	40.3	24.9	103.5	566.6	107.9	5.5	55.5	169.1	539.0	1420.2	2450.7
HIV21	HAD	GTS <40	2.7	149.0	241.7	8.8	556.4	14.1	13.3	36.6	37.8	50.5	22.0	41.8	10.3	170.9	627.6	113.4	8.0	40.8	145.6	842.0	1415.3	1661.5
HIV22	HAD	GTS <40	2.5	175.8	244.7	5.5	257.6	9.5	6.5	28.7	22.8	29.6	14.1	42.2	10.1	73.2	414.5	27.8	4.7	33.9	140.6	384.2	856.7	1445.9
HIV23	ANI	GTS <40	1.8	158.2	127.4	5.6	522.4	9.1	5.8	39.1	16.8	36.8	16.1	28.5	18.9	84.8	497.4	96.8	3.3	27.6	143.2	546.2	957.4	2173.9
HIV24	NPI-O	GTS <40	2.5	218.3	293.7	4.4	420.5	6.7	4.4	42.7	14.8	19.6	7.5	46.4	9.4	51.3	277.1	27.8	5.1	26.0	208.9	382.8	634.1	1309.2
HIV25	MND	GTS <40	2.7	109.3	408.9	3.7	166.5	6.4	2.7	18.2	14.1	43.6	11.1	59.2	15.4	43.6	1002.6	355.8	6.4	171.7	148.6	184.2	5052.9	11444.6
HIV26	NPI-O	GTS <40	2.8	168.5	116.9	7.3	1131.2	11.9	7.7	137.5	30.5	35.3	12.7	25.6	11.9	114.8	626.7	19.5	4.9	51.2	311.1	749.5	1233.0	778.7
HIV27	MND	GTS <40	2.7	105.6	89.2	19.5	1453.2	31.5	36.1	97.8	95.2	101.9	68.5	110.9	17.4	459.7	1425.9	76.2	16.8	59.8	256.5	2265.2	1188.1	1174.6
HIV28	NPI-O	GTS <40	3.0	193.6	346.0	5.7	241.0	8.9	6.2	13.7	18.6	33.0	11.7	29.2	10.3	69.1	644.9	154.8	6.1	49.8	117.5	368.3	8058.6	8700.5
HIV29	HAD	GTS <40	1.9	141.3	215.2	3.4	197.2	7.0	2.5	22.8	12.9	23.5	2.7	13.6	10.3	43.8	381.1	113.4	4.1	57.1	89.5	270.1	1905.1	1774.2
HIV30	MND	GTS <40	2.1	96.7	222.2	1.8	81.1	3.0	2.1	3.7	4.2	14.4	1.5	2.9	5.9	11.3	104.9	59.8	1.4	57.5	75.6	85.2	1008.9	2459.4
Ctrl1		Control	1.9	58.2	28.0	4.1	198.4	5.2	1.2	17.0	28.5	22.5	2.5	16.4	2.1	54.9	368.0	121.1	7.0	140.7	66.6	220.8	1557.4	6617.0
Ctrl2		Control	1.7			3.9	179.7	7.3	2.1	12.6	36.0	33.5	5.9	17.1	1.0	37.8	192.4	362.0	3.2	241.8	63.5	243.7	1458.5	2001.6
Ctrl3		Control	1.6	128.1	58.7	2.7	161.0	5.8	2.1	14.5	27.9	26.6	0.8	9.2	51.4	11.3	105.0	198.0	7.7	171.1	118.0	314.6	323.3	1865.0
Ctrl4		Control	1.6	6.6	16.4	3.8	200.7	6.4	0.8	13.3	23.0	31.6	8.7	27.9	5.2	40.4	158.1	210.9	6.5	217.2	69.7	221.0	675.5	1222.9
Ctrl5		Control	1.6	7.1	19.1	3.7	201.8	6.5	0.9	14.2	21.1	33.2	12.8	30.4	5.0	36.0	158.1	103.4	8.9	225.8	69.8	224.8	632.4	1190.9
Ctrl6		Control	1.8	45.7	122.8	3.1	101.1	3.3	2.1	3.7	17.9	21.6	0.8	2.9	0.9	30.6	96.5	223.8	6.8	186.7	36.7	76.6	399.8	953.7
Ctrl7		Control		137.9	62.4	2.3	137.4	4.3	2.1	9.1	22.6	21.3	1.5	6.5	6.3	11.3	60.8		7.0	150.5	87.1	224.7	259.5	1662.0
Ctrl8		Control	1.7	63.2	171.1	1.8	26.1	1.6	2.2	7.2	3.2	8.4	0.7	6.2	2.7	10.3	153.7	72.3	1.4	66.2	56.8	36.5	706.5	1589.4
Ctrl9		Control	1.3	66.5	9.7	2.1	40.4	1.8	2.2	2.8	3.1	7.8	3.7	5.1	6.3	16.4	266.0	31.3	1.4	45.5	88.5	38.1	490.6	864.8
Ctrl10		Control	1.7	65.2	40.4	1.8	38.3	2.2	0.2	1.8	5.0	6.6	3.3	1.2	4.5	13.5	173.4	66.2	1.4	54.7	34.3	59.8	646.3	1160.3
Ctrl11		Control	1.3	114.3	23.8	2.2	63.3	2.4	1.2	2.7	5.8	15.4	8.1	2.4	2.7	18.6	101.5	66.2	3.2	69.9	34.3	65.9	642.3	1112.3
Ctrl12		Control	1.1	77.2	26.9	1.6	38.3	2.4	2.2	2.0	5.4	7.2	2.7	1.5	5.3	13.5	54.3	31.3	1.4	52.2	34.3	56.7	355.8	1206.7
Ctrl13		Control	2.3	90.9	240.0	2.7	94.2	5.1	0.5	8.7	8.2	17.8	4.7	3.8	16.8	25.1	224.0	72.3	1.2	65.3	145.7	151.7	335.1	1388.8
Ctrl14		Control	2.2	57.4	23.3	2.7	71.7	5.5	2.2	10.2	5.4	19.8	6.2	2.8	17.7	27.8	397.7	75.4	1.4	169.8	185.2	118.9	895.6	1095.5
Ctrl15		Control	1.2	50.8	35.7	2.8	106.2	4.6	2.2	15.2	9.0	15.2	5.0	3.6	6.1	21.9	301.6	31.3	3.9	35.0	113.5	102.4	722.9	692.6
Ctrl16		Control	1.1	33.7	42.2	2.2	44.5	2.2	2.2	4.7	3.6	9.0	5.3	6.5	6.8	13.5	180.0	118.6	1.4	66.2	103.3	61.3	776.9	1654.3
Ctrl17		Control	1.0	64.6	20.7	3.4	180.2	4.8	2.2	18.4	3.0	14.4	3.8	3.6	9.6	29.7	368.5	124.8	1.4	47.5	178.0	165.0	677.3	911.7
Ctrl18		Control	1.7	55.8	115.5	2.0	39.3	2.5	2.2	3.7	3.6	7.3	3.2	2.4	4.5	16.0	272.7	177.2	1.4	54.8	66.4	50.5	593.9	2622.0
Ctrl19		Control	1.3	79.1	99.7	1.8	41.4	2.3	2.2	3.7	3.7	8.5	5.0	3.6	2.2	11.9	132.9	103.2	1.4	56.1	63.4	36.5	365.6	487.2
Ctrl20		Control	1.5	82.2	78.8	1.7	86.7	2.3	2.2	11.6	3.4	9.2	12.6	10.2	2.1	10.9	375.3	121.7	1.4	92.0	124.7	64.4	413.3	2118.9

		sCD14	HA	YKL-40	IL-1b	IL-1RA	IL-4	IL-5	IL-6	IL-7	IL-8	IL-10	IL-12	IL-13	TNF-alpha	sIL-2R	CCL2	CCL3	CCL4	IFN-alpha	IFN-gamma	CXCL9	CXCL10
**Average**	**Controls**	1.6	67.6	65.0	2.6	102.5	3.9	1.8	8.8	12.0	16.8	4.9	8.2	7.9	22.6	207.0	121.6	3.5	110.4	87.0	126.7	646.4	1620.9
	**GTS ≥40**	2.3	138.0	207.0	6.3	364.1	11.8	7.9	25.6	26.3	37.2	19.1	46.0	12.6	103.3	504.2	165.4	6.4	61.4	139.2	518.6	2488.5	4358.2
	**GTS <40**	2.5	163.8	247.0	7.0	486.1	12.1	9.8	42.5	29.2	40.9	17.4	38.5	13.9	116.0	650.2	136.5	6.3	56.7	166.7	609.9	4704.0	4027.8
**Median**	**Controls**	1.6	64.6	40.4	2.5	90.4	3.8	2.2	8.9	5.6	15.3	4.2	4.4	5.1	17.5	176.7	103.4	1.4	68.0	69.8	89.5	637.3	1214.8
	**GTS ≥40**	2.4	117.4	181.5	6.3	329.5	12.0	8.4	25.7	26.6	39.9	15.6	37.8	12.1	97.0	487.6	163.2	5.6	57.9	134.7	515.1	1629.3	2979.0
	**GTS <40**	2.5	158.2	241.7	5.7	401.4	9.5	7.5	36.6	22.8	36.8	14.1	40.3	11.9	84.8	627.6	107.9	5.5	54.6	148.6	482.5	1415.3	2173.9

		sCD14	HA	YKL-40	IL-1b	IL-1RA	IL-4	IL-5	IL-6	IL-7	IL-8	IL-10	IL-12	IL-13	TNF-alpha	sIL-2R	CCL2	CCL3	CCL4	IFN-alpha	IFN-gamma	CXCL9	CXCL10
**Kruskal-Wallis test**		< 0.0001	< 0.0001	< 0.0001	< 0.0001	< 0.0001	< 0.0001	< 0.0001	< 0.0001	0.0012	< 0.0001	< 0.0001	< 0.0001	0.0003	< 0.0001	< 0.0001	0.2256	0.0075	0.0140	0.0004	< 0.0001	< 0.0001	0.0030
**Mann-Whitney with Bonferroni Adjustment***	**GTS ≥40 vs. Control**	2.80E-04	7.70E-06	1.70E-04	3.30E-06	6.80E-06	3.30E-06	4.80E-06	2.10E-05	1.20E-03	6.30E-05	1.70E-05	6.20E-06	4.60E-04	1.90E-06	3.80E-05	8.34E-02	3.20E-03	3.43E-02	3.17E-03	2.60E-06	5.50E-06	2.60E-03
	**GTS <40 vs. Control**	6.20E-06	4.00E-07	7.40E-07	6.20E-05	3.80E-05	1.50E-05	3.40E-06	1.70E-05	3.90E-03	9.60E-05	3.80E-04	7.20E-05	9.00E-04	2.10E-05	2.10E-05	7.81E-01	2.58E-02	8.40E-03	4.90E-04	2.50E-05	1.00E-04	8.10E-03
	**GTS ≥40 vs. GTS <40**	4.79E-01	1.70E-01	3.45E-01	1.00E+00	7.10E-01	6.90E-01	9.20E-01	8.90E-02	8.03E-01	9.00E-01	5.47E-01	5.30E-01	8.03E-01	6.90E-01	1.50E-01	2.30E-01	6.19E-01	4.36E-01	2.33E-01	1.00E+00	9.00E-01	7.13E-01

**pvalue legend**
**>0.05**
**>0.017 and ≤0.05**
**>0.001 and ≤0.017**
**>0.0001 and ≤0.001**
**<0.0001**

* Bonferroni adjusted p-value threshold p<0.017; GTS, global T score; Normal, No Impairment, no neurocognitive impairment; ANI, asymptomatic neurocognitive impairment; MND, Mild Neurocognitive Disorder; HAD, HIV-associated dementia; NPI-O, Neuropsychological impairment due to other causes.
