# Supplementary material for: Applications and Limitations of Inflammatory Biomarkers for Studies on Neurocognitive Impairment in HIV Infection
Source: J Neuroimmune Pharmacol. 2013 Nov 21;8(5):1087–97. doi: 10.1007/s11481-013-9512-2 (PMC3889222; doi:10.1007/s11481-013-9512-2)
Supplement: Supplementary file 4 — P-values and false discovery rates (FDR) for between-group comparisons of plasma inflammatory biomarker levels in HIV+ subjects classified by neurocognitive status one year after the baseline visit. Based on HAND clinical diagnoses and global T scores after one year follow-up compared to the baseline visit, HIV+ subjects were classified into dichotomous groups with no/improved neurocognitive impairment (NCI) (n=7) or stable/worse NCI (n=16) at one year follow-up as described in the Supplemental Methods. A third group consisted of HIV-/HCV- healthy controls (n=20). These 7 inflammatory biomarkers represent a cluster of biomarkers that correlated positively in pair-wise Pearson correlations in exploratory analyses of 22 biomarkers (see Supplemental Figure 1). Significant differences were defined by a p-value <0.05 and controlling the FDR at < 5%. FDR was calculated in R using p.adjust. (PDF 10 kb) [file 11481_2013_9512_MOESM4_ESM.pdf]

Supplemental Table 3. P-values and false discovery rates (FDR) for between-group comparisons of plasma inflammatory biomarker levels in HIV+ subjects classified by neurocognitive status one year after the baseline visit. HIV+ subjects were classified into dichotomous groups with no/improved NCI (n=7) or stable/worse NCI (n=16) at one year follow-up as described in the Supplemental Methods. A third group consisted of HIV-/HCV- healthy controls (n=20). These 7 inflammatory biomarkers represent a cluster of biomarkers that correlated positively in pair-wise Pearson correlations in exploratory analyses of 22 biomarkers (Supplemental Figure 1). Significant differences were defined by a p-value <0.05 and controlling the FDR at < 5%. FDR was calculated in R using p.adjust.

	**Control vs. No NCI/IMPROVED**		**Control vs. Stable/Worse NCI**		**No NCI/IMPROVED vs. Stable/Worse NCI**	
**Analytes**	**p-value**	**FDR**	**p-value**	**FDR**	**p-value**	**FDR**
IFN-alpha	0.0913	0.0913	2.70E-08	9.45E-08	0.0045	0.0315
IL-1b	0.0093	0.0130	2.80E-07	3.92E-07	0.0766	0.1072
IL-6	0.0031	0.0065	1.00E-06	1.17E-06	0.0101	0.0354
IL-12	0.0173	0.0202	2.90E-06	2.90E-06	0.0569	0.0996
IFN-gamma	0.0012	0.0065	1.00E-07	2.33E-07	0.3326	0.3326
sIL-2R	0.0037	0.0065	2.70E-08	9.45E-08	0.0212	0.0495
IL-1RA	0.0021	0.0065	1.90E-07	3.33E-07	0.1164	0.1358
