# Supplementary material for: Applications and Limitations of Inflammatory Biomarkers for Studies on Neurocognitive Impairment in HIV Infection
Source: J Neuroimmune Pharmacol. 2013 Nov 21;8(5):1087–97. doi: 10.1007/s11481-013-9512-2 (PMC3889222; doi:10.1007/s11481-013-9512-2)

Pearson Scatterplot Matrix

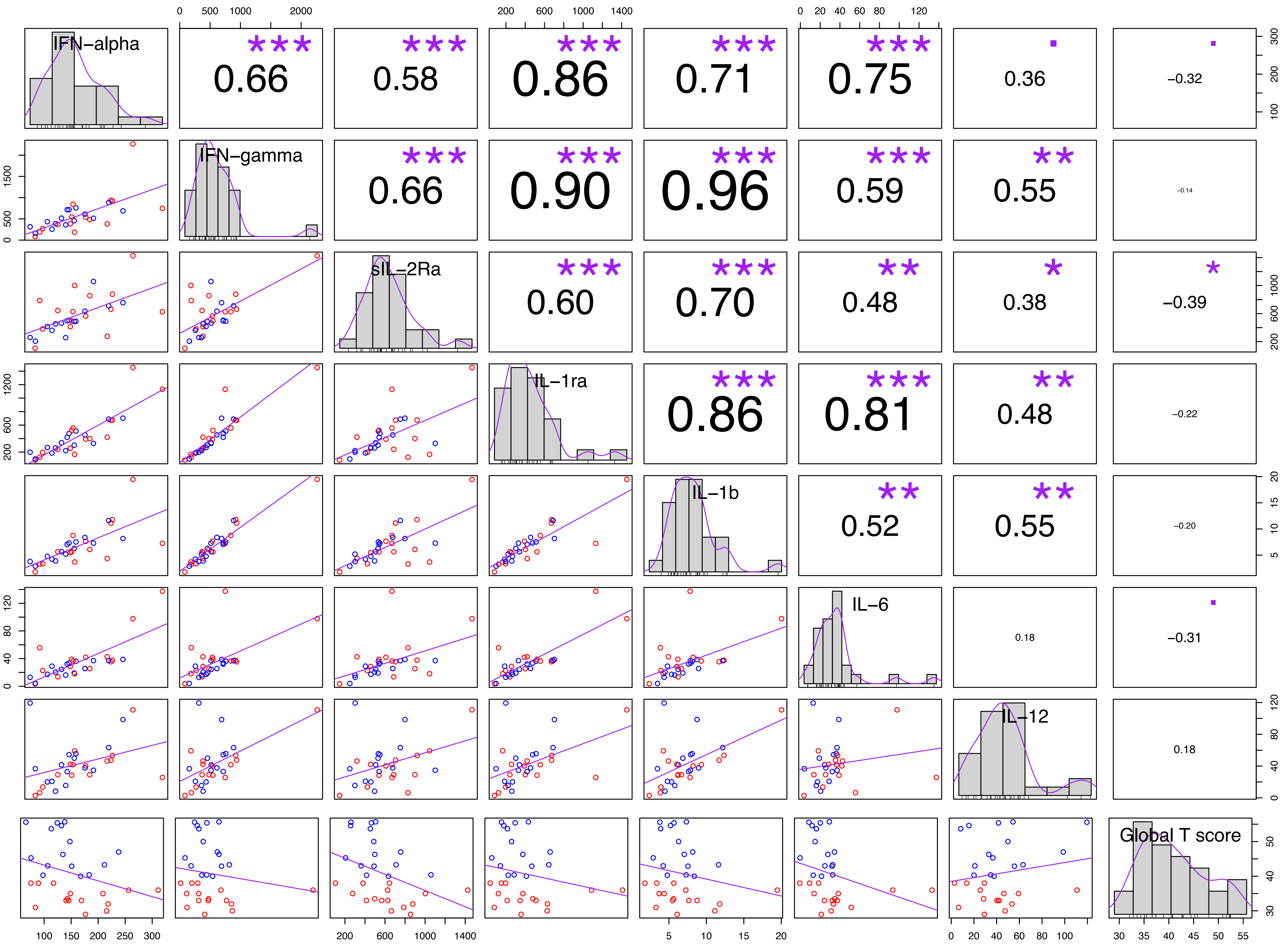

Supplement: Supplementary file 5 — Pearson correlation matrix visualization reveals inter-relationships between inflammatory biomarkers and global T-scores in HIV+ subjects with advanced disease on suppressive ART. Inflammatory biomarkers were compared in a pair-wise manner and the correlation matrix was constructed in R. Diagonals display the histogram, kernel density curve (purple), and rug for each individual biomarker. Panels to the right of the histogram show Pearson correlation coefficients. The text is scaled relative to the absolute magnitude of the correlation coefficient. P values are represented by purple symbols (*** p<0.001; ** p<0.01; * p<0.05; • p= 0.05-0.1). Panels left of the diagonal show scatterplots. The linear regression line (purple) fits expression values from all 30 HIV subjects. Red and blue circles represent HIV subjects with T scores <40 or ≥ 40, respectively. (PDF 565 kb) [file 11481_2013_9512_MOESM5_ESM.pdf]
